# Identification of PilD mutants reveals the iterative social evolution in *Pseudomonas aeruginosa*

**DOI:** 10.1128/aem.00915-25

**Published:** 2025-09-04

**Authors:** Huifang Qiu, Xiaoqing Zhou, Weijun Dai

**Affiliations:** 1 Integrative Microbiology Research Center, College of Plant Protection, South China Agricultural University 673803, Guangzhou, China; Norwegian University of Life Sciences, Ås, Norway

**Keywords:** quorum sensing, PilD, *Pseudomonas aeruginosa*, experimental evolution

## Abstract

Bacterial pathogenicity is influenced by the genetic mutations that arise within the population, among which quorum-sensing (QS) is one of the key targets. Recent studies have reported that mutations in the global transcriptional regulator gene *mexT* can revert QS activity in QS-inactive LasR mutants, thereby transforming the LasR cheater mutants deficient in public goods production into cooperators. However, the subsequent evolutionary trajectory of this population remains poorly understood. To gain a deeper understanding of the evolutionary trajectory of *Pseudomonas aeruginosa*, we conducted an *in vitro* evolution experiment with the LasR-MexT-deficient mutant strain and examined the emerging variant subpopulations. Under the condition of QS activation, we identified a variant subpopulation carrying mutations in *pilD*, which encodes a prepilin peptidase involved in both type II secretion system (T2SS) and type IV pili (T4P). In contrast to the parental cooperative LasR-MexT mutant, the PilD mutant displays a social cheater phenotype, characterized by a defect in extracellular protease secretion. The inactivation of PilD resulted in reduced QS activity and the production of QS-controlled products. Furthermore, the PilD mutant caused significantly reduced cell cytotoxicity in mammalian cells. Our findings highlight an iterative evolution of social behavior between cooperators and cheaters. In conclusion, our study provides a roadmap of *P. aeruginosa*’s evolutionary trajectory in the QS activation environment, shedding light on our understanding of the evolutionary dynamics underlying bacterial pathogenicity.

**IMPORTANCE:**

*P. aeruginosa* is a leading cause of opportunistic acute and chronic infections in humans, in which its pathogenicity is intricately intertwined with its evolutionary trajectory and the emergence of genetic mutants within the population. Our studies reveal an iterative social development between cooperative and cheating behaviors, providing valuable insights into the intricate dynamics of social interactions within bacterial populations. Furthermore, our investigations demonstrate dynamic mutant pathogenicity changes during the evolutionary process, suggesting that developing strategies to combat antibiotic resistance and pathogenicity in clinical settings.

## INTRODUCTION

*Pseudomonas aeruginosa* is an opportunistic pathogen that causes severe acute and chronic human infections, particularly in individuals with compromised immune systems, such as those with cystic fibrosis (CF) (1, 2). *P. aeruginosa* has a cohort of virulence factors that are controlled by the quorum-sensing (QS) system. QS is a bacterial cell-cell communication mechanism that regulates the expression of hundreds of genes in a cell density-dependent manner (3). Two acyl-homoserine lactone (AHL) QS systems, Las (LasI and LasR) and Rhl (RhlI and RhlR), have been identified in *P. aeruginosa*. LasI and RhlI catalyze the biosynthesis of diffusible QS signal N-3-oxododecanoyl homoserine lactone (3OC12-HSL) and butyryl-HSL (C4-HSL), respectively. Upon binding with 3OC12-HSL, LasR activates the expression of genes in the Las-regulon. Similarly, the C4-HSL-bound RhlR induces the expression of genes in the Rhl-regulon. Additionally, these two AHL QS systems also interact with a third QS system, known as the PQS system, which involves the production of 2-heptyl-3-hydroxy-4-quinolone (PQS) and its corresponding signal receptor PqsR (also referred to as MvfR). In general, QS systems in *P. aeruginosa* are organized hierarchically, with the Las QS system positioned at the top of the QS hierarchy. Inactivation of either LasI or LasR leads to the inactivation of all three QS circuits in the standard laboratory strain PAO1 (3, 4).

*P. aeruginosa*, when invading host cells, typically thrives in hostile and stressful environments, including host immune responses, exposure to antibiotics, and competition with other microorganisms (5–7). Consequently, *P. aeruginosa* has to undergo extensive evolutionary adaptations to persist and survive within these conditions (8, 9). Genome sequencing analyses have revealed that adaptive mutations frequently occurred in the genomes of longitudinal isolates obtained from CF patients. These genetic mutations fall into various functional categories, including virulence, motility, transport, antibiotic resistance, iron acquisition, DNA replication, and regulatory systems. A significant proportion of these mutations affects genes encoding key global regulators, such as *lasR, rpoN, mexT, gacS, retS, exsD,* and *ampR* (10–15). Notably, *lasR* mutants (QS signal-blind) were predominantly isolated in clinical acute and chronic infections of *P. aeruginosa* (10, 16, 17). These mutants frequently possessed loss-of-function mutations (18), which are important for the evolutionary adaptation of *P. aeruginosa* to changing host environments.

QS signal-blind mutants, such as LasR mutants, were found to be more common in clinical *P. aeruginosa* isolates than signal-negative mutants, like LasI mutants (10, 16, 17). These QS signal-blind mutants can be considered social cheaters within the population, since they no longer produce extracellular public goods, whereas QS signal-negative mutants continue to contribute to the community by responding to QS signals secreted by surrounding cells (19, 20). In addition to clinical contexts, QS signal-blind mutants have also been reported to emerge within the population under protein-based evolution conditions, in which QS activation is necessary for secreting QS-controlled proteases for cell growth. Using this approach, LasR and PqsR mutants have been identified and characterized as social cheaters (21–25). These two typical cheater mutants exhibited defective QS-controlled public goods (21), whereas interestingly, the RhlR mutant was not found in this context (21). Moreover, the social cheating role of the LasR mutant was reinforced in a mouse infection model (26). These findings provide an explanation for the enrichment of QS signal-blind mutants in QS-dependent environments and suggest that some infection mutants are selected based on their social roles.

Mutations in the *mexT* gene have been observed in various environments, including *P. aeruginosa* isolates from CF patients (10), intestinal tissues (27), and laboratory strain PAO1 (28, 29). MexT encodes a transcriptional regulator belonging to the LysR family and positively controls the expression of the *mexEF-oprN* operon genes (30, 31). In addition to the *mexEF-oprN* operon, MexT also globally regulates the expression of more than 40 genes (32). As the MexEF-OprN pump is responsible for the transport of the PQS precursor HHQ, MexT also regulates QS-dependent activities (33). Inactivation of MexT results in the increased production of pyocyanin, enhanced swarming motility, reduced chloramphenicol resistance, and elevated destructive capability on tissues (27, 28). Through *in vitro* evolution experiments with the laboratory strain PAO1, mutations in *mexT* were found to be capable of reverting Rhl QS activity in the LasR-null strain (34, 35). However, the subsequent evolutionary trajectory of the LasR-null QS revertant remains unexplored.

In the present study, we conducted an evolution experiment with a constructed PsdR-LasR-MexT-deficient mutant of *P. aeruginosa* strain PAO1 under the conditions that require QS activation. Three independent mutant lines were evolved, and protease-negative colony mutants were screened. Our findings revealed that mutants with mutations in the *pilD* gene exhibited a social cheating behavior, characterized by deficient secretion of extracellular proteases . Moreover, the deletion of *pilD* resulted in reduced activities of both Rhl and PQS QS systems, leading to a decrease in the production of QS-controlled factors. Additionally, the PilD mutant showed reduced cytotoxicity toward host mammalian cells. By integrating previous findings and our results, our study reveals that *P. aeruginosa* undergoes iterative social evolution with QS variant mutants alternating between cooperation and cheating, resulting in dynamic changes in mutant virulence. Our study provides valuable insights into the intricate dynamics of social interactions and phenotypic consequences within a bacterial population. Moreover, these findings link genetic changes to evolutionary trajectories and highlight the importance of understanding the evolution of pathogens in the context of chronic infections.

## MATERIALS AND METHODS

### Bacteria and growth conditions

*P. aeruginosa* strain PAO1 and derivatives were grown in Luria-Bertani (LB) broth or LB broth buffered with 50 mM 3-(N-morpholino) propanesulfonic acid, pH 7.0 (LB-Mops broth) at 37°C. In some experiments, *P. aeruginosa* were cultured in M9 medium (36) containing 1% sodium caseinate (C8654, Sigma-Aldrich, New Zealand) (M9-casein) as the sole source of carbon and energy for evolution experiments or 0.5% casamino acids (35) (A100851, Sangon Biotech, Shanghai, China) (M9-CAA) at 37°C. *Escherichia coli* was grown in LB broth at 37°C. Unless otherwise specified, *P. aeruginosa* strains were cultured in 14-mL Falcon tubes containing 3 mL medium at 37°C with shaking at 220 rpm. Colonies were grown in LB agar or *Pseudomonas* Isolation agar (PIA) (1.5% agar). Details of bacterial strains used in this study are listed in Table S3.

### Construction of *P. aeruginosa* PAO1-derivative mutants

Construction of *P. aeruginosa* PAO1 mutants (PsdR-LasR-MexT and PsdR-LasR-MexT-PilD) was performed using a homologous recombination approach as described previously (37). Briefly, approximately 500–1,000 bp DNA flanking the full-length target gene was amplified and cloned into the pGEX2 vector using the Vazyme CloneExpress II One Step Cloning Kit (Vazyme Biotech, Nanjing, China). The generated pGEX2-flanking constructs (100 µg/mL gentamycin) were mobilized into *P. aeruginosa* strains by triparental mating with the help of pRK2013 (50 µg/mL kanamycin). Mutants were first selected on PIA plates supplemented with 100 µg/mL gentamycin and then counter-selected on LB agar containing 10% (wt/vol) sucrose. All mutants were confirmed by PCR amplification and subsequent Sanger DNA sequencing. The primers used in this study are listed in Table S4.

### Evolution experiment

Three independent colonies of assayed strains were separately inoculated into 3 mL LB-Mops broth overnight. Evolution experiments were initiated by transferring 100 µL overnight cultures into 3 mL M9-casein at 37°C for 36 h. After that, evolution was carried out every day by transferring 30 µL bacterial suspension into 3 mL fresh M9-casein medium. The protease variant mutants were determined by spreading clones on LB agar plates and spotting single colonies onto the skim milk plates at 37°C for 18–24 h.

### Complementation of the PsdR-MexT-PilD mutant

The *pilD* gene fused with an *E. coli* ribosomal *rrnB* P1 promoter (38) was cloned into the plasmid pUC18T-mini-Tn7T-Gm (39) with the Vazyme Clone Express II One Step Cloning Kit, generating miniTn7-P*rrnB-pliD* (100 µg/mL gentamycin). The constructed miniTn7-P*rrnB-pliD* was integrated into the neutral *att* site of the PAO1 genome by electroporation transformation with the assistance of a helper plasmid pTNS2. Genomic integration of miniTn7-P*rrnB-pilD* was confirmed by PCR amplification and subsequent Sanger DNA sequencing.

### Skim milk assay

Total extracellular proteolytic activity of *P. aeruginosa* strains was evaluated using skim milk agar plates (25% [vol/vol] LB, 4% [wt/vol] skim milk, and 1.5% [wt/vol] agar). An equal amount of overnight cultured monoclonal bacteria was spotted on the plate and grown at 37°C for 18–24 h. Colonies with extracellular proteolytic activities formed clearing zones and were photographed.

### Twitching motility assay

PAO1 derivatives were grown overnight at 37°C on LB plates. Equal amounts of bacteria were spotted onto the bottom of LB plates (1% agar) with toothpicks. Bacteria were incubated at 37°C for 24 h. The agar was then removed, and the twitching zone was visualized by staining with 1% crystal violet for 10 min and washing with distilled water.

### QS reporter assay

For the construction of the QS reporter plasmids, DNA fragments spanning 533 bp, 322 bp, and 558 bp upstream of the *rhlA*, *pqsA,* and *lecB* genes were amplified and cloned into the pPROBE-GT vector, respectively. This generated the transcriptional fusion reporters P*rhlA*-GFP, P*pqsA*-GFP, and P*lecB*-GFP. All primers used for QS reporter constructions are listed in Table S4. QS reporter plasmids were transferred into PAO1 derivatives by triparental mating, and colonies were selected on a PIA plate (100 µg/mL gentamycin). For the QS expression assay, PAO1 strains bearing QS reporter plasmids were cultured in LB-Mops broth containing 100 µg/mL gentamycin overnight. The bacterial suspension was diluted to M9-CAA containing 100 µg/mL gentamycin (OD_600_ ≈ 0.01) and transferred into a 96-well plate (200 µL/well) with at least six technical replicates. Fluorescence (excitation 488 nm, emission 525 nm) and optical density (OD_600_) values of the samples were determined using a microplate reader machine (Synergy H1MF, BioTek Instruments, Winooski, VT, USA).

### Elastase production

*P. aeruginosa* strains grown in LB broth overnight were diluted to 3 mL fresh LB broth with an OD_600_ ≈ 0.01 and grown with shaking at 37°C for 12 h. Cells were precipitated, and 500 μL supernatant was transferred to a tube containing the same amount of ECR (Elastin-Congo red) buffer (0.1M Tris-HCl, 1 mM CaCl2, 5 mg/mL Elastin-Congo red [E0502, Sigma-Aldrich, USA], pH 7.2) and incubated at 37°C for 2 h. The reaction was stopped on ice using 100 µL EDTA (0.12 M). The insoluble ECR was pelleted at 5,000 × g for 5 min at 4°C, and the supernatant was determined by measuring OD_495_.

### Pyocyanin measurement

Overnight cultures of *P. aeruginosa* grown in LB broth were diluted into 4 mL M9-CAA medium to reach an initial OD_600_ ≈ 0.01. The diluted bacteria were grown at 37°C for 24 h. Cells were collected at 13,000 rpm for 2 min. The supernatants were measured at OD_695_, and bacterial solutions were measured at OD_600_. Pyocyanin production was estimated by determining OD_695_/OD_600_.

### Biofilm production

*P. aeruginosa* strains were cultured in LB broth overnight and then diluted into fresh LB broth (OD_600_ ≈ 0.01). The bacteria were grown to reach mid-log phase (OD_600_ ≈ 0.4–0.6) at 37°C. The mid-log phase cultures were then diluted with fresh LB broth (OD_600_ ≈ 0.01) and transferred into the polystyrene 96-well plates (200 μL/well) and incubated at 37°C for 24 h. The unattached cells in the 96-well plates were gently rinsed with 230 μL distilled water, and the attached cells (biofilms) were stained with 250 μL 0.1% crystal violet at room temperature for 10 min. Unstained crystal violet was removed by gently washing with 275 μL distilled water. The biofilms were suspended in 300 μL 95% ethanol at room temperature for 10 min and quantified by measuring the absorbance at 595 nm. Each experiment was repeated at least three times.

### Mammalian cell cytotoxicity assay

Chinese hamster ovary (CHO) cells (AcroImmune Group, Guangzhou, China) were seeded in 96-well plates (1.5 × 10^4^ cells/well) containing 100 µL RPMI medium (Thermo Fisher Scientific, Shanghai, China) supplemented with 10% FBS (Thermo Fisher Scientific, Shanghai, China) and incubated at 37°C with 5% CO_2_ to obtain 80%–90% monolayer confluency. Exponentially growing *P. aeruginosa* strains cultured in LB broth (OD_600_ ≈ 0.5) were diluted with RPMI medium supplemented with 1% FBS (OD_600_ ≈ 0.1) and added to CHO cells at a starting multiplicity of infection (MOI) ratio of 5:1. After incubation at 37°C for 6 h, the extent of cell killing was determined by quantification of the release of lactate dehydrogenase (LDH) in the cell culture supernatant with the LDH Cytotoxicity Detection Kit (Beyotime, Nantong, China).

### Competition experiment

Bacteria were grown in LB-Mops broth overnight. The overnight cultured bacteria were then diluted with LB-Mops broth to yield an initial OD_600_ of 0.01. These bacteria were further grown to OD_600_ ≈ 3.5–4.0. The constructed PsdR-LasR-MexT mutant was then mixed with the PsdR-LasR-MexT-PilD mutant at a ratio of 90:10 (900 µL PsdR-LasR-MexT and 100 µL PsdR-LasR-MexT-PilD). A competition experiment was subsequently performed by transferring 75 µL bacterial mixture into 3 mL fresh M9-casein broth. Bacteria were incubated at 37°C and passaged every 24 h. The colonies were counted on skim milk agar plates every 2 days or daily (if PsdR-LasR-MexT-PilD increases rapidly within the population, indicated by the color of the bacterial culture tube).

### Whole genome sequencing by Illumina HiSeq

In total, 1 µg of microbial genomic DNA was sonicated to an average size of ~ 350 bp by the Covaris-S220 ultrasonicator (Covaris, Woburn, MA, USA). Illumina DNA fragment library preparation was performed following the manual of the next-generation sequencing DNA library preparation kit (Novagen). Briefly, the fragmented DNA products were end-repaired and ligated with an adapter. Adapter-ligated products were purified using AMPure XP beads (Agencourt-Berkman Coulter, USA) and enriched through PCR amplification using the custom adapter-specific primers. The obtained unbiased short-read library was further cleaned up with AMPure XP beads. Pair-end Illumina HiSeq PE150 sequencing was performed with an Illumina Novaseq 6000 sequencing system.

### Analysis of Illumina HiSeq short reads

Raw short reads were subjected to quality control, including removing adapters using Cutadapt by Novagen (Novagen, Tianjin, China), yielding clean short reads. Clean short reads were mapped to the *P. aeruginosa* PAO1 reference genome (NC_002516.2) with Burrows-Wheeler Alignment (BWA) software. The mapped short reads were subjected to a genome-wide genetic variant calling using Samtools and customized Perl scripts.

### Software

The following software was used in this study: BWA software, version 0.7.15-r1140 (http://bio-bwa.sourceforge.net) (40); Cutadapt software, version 1.16 (https://cutadapt.readthedocs.io/en/v1.16/) (41); Samtools software, version 1.5 (http://samtools.sourceforge.net) (42); FastQC, version fastqc_v0.11.5 (https://www.bioinformatics.babraham.ac.uk/projects/fastqc/); Hisat2, version 2.1.0 (https://daehwankimlab.github.io/hisat2/) (43); HTSeq, version 0.11.1 (https://htseq.readthedocs.io/en/release_0.11.1/count.html) (44); DESeq2 (45); Perl software, version v5.22.1 (https://www.perl.org/); Python software, version v3.8.2 (https://www.python.org/downloads/release/python-382/); R software, version v3.6.1 (http://www.R-project.org/); GraphPad Prism software, version 5 (https://www.graphpad.com/); CorelDRAW software, version 2020 (https://www.coreldraw.com/en/).

### Statistical analysis

Statistical analyses were performed using Excel and GraphPad Prism 5 (https://www.graphpad.com/).

## RESULTS

### Continued evolution of the LasR-MexT mutant to investigate the population evolutionary trajectory

As the cooperator LasR-MexT mutant persists under QS-dependent selection pressures, such as casein-based broth, we hypothesized that this mutant would continue to evolve, differentiating into unknown variant mutants. As a test of our hypothesis, we performed a preliminary evolution experiment starting with a QS-inactive LasR-null mutant of *P. aeruginosa* strain PAO1. This protease-negative mutant was subjected to evolution in a casein-based broth, a medium in which QS activation is closely related to the production of QS-controlled extracellular proteases necessary for cell growth (22, 23). The protease activity of mutant colonies was assessed through the skim milk agar plate assay. Protease-positive mutant colonies were visible in the skim milk agar plates by day 15 (Fig. S1). Consistent with previous findings (34, 35), we identified mutations in *mexT* in these protease-positive mutant colonies. Evolution of the same population from day 15, which already contained *mexT* mutants, led to the discovery of protease-negative mutant colonies carrying the same mutated *mexT* by day 30 (Fig. S1), implying the involvement of unknown determinants responsible for this protease-revertant phenotype. To identify the occurring genetic changes, two such mutant colonies were submitted to whole-genome sequencing (WGS). WGS analysis revealed a deletion in *psdR* and single-nucleotide substitutions in *mexT*, *pilD,* and some non-coding regions in the analyzed LasR-null strain (Table S1). Given that PsdR regulates the uptake and transport of the dipeptide (a molecule consisting of two amino acids linked by a peptide bond) (46, 47) and regulates QS through *lasR* transcription (48), its involvement in this protease-revertant phenotype of the LasR-MexT mutant seems very unlikely. The *pilD* gene seemed to be a candidate requiring further verification. PilD encodes a prepilin peptidase crucial for the functionality of both type II secretion system (T2SS) and type IV pili (T4P) (49–51). PilD-disrupted mutants have been shown to result in the accumulation of enzymes, including alkaline phosphatase and elastase, in the periplasmic spaces (52). In conclusion, our preliminary experiment uncovers a previously unreported evolutionary trajectory, illustrating that the LasR-MexT QS revertant mutant further evolves into protease-negative mutants through the acquisition of secondary adaptive mutations.

### Identification of mutations in *pilD* responsible for the protease-deficient phenotype

**Fig 1:**
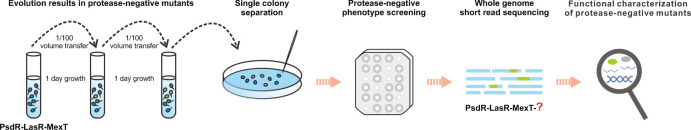
Diagram illustrating the evolution experiment. The overall procedure of the evolution experiment developed in our study. The evolution experiment is initiated with a PsdR-LasR-MexT mutant. The bacteria are passaged daily with casein-based broth and spread to skim milk agar plates to screen for mutants with altered phenotypes. This experiment results in the identification of protease-negative mutants.

To recapitulate the evolutionary trajectory described above, we repeated the evolution experiments using an engineered PsdR-LasR-MexT mutant. The *psdR* gene, commonly mutated under the casein-based evolution condition, was deleted from the LasR-MexT mutant (35, 36, 53). As a starting point, we clarified whether the constructed PsdR-LasR-MexT mutant harbored secondary mutations introduced during its creation. WGS analysis conclusively indicated that no additional genetic mutations could be detected in the PsdR-LasR-MexT mutant under the sequencing conditions with whole-genome coverage (100%) by mapping short reads and rather deep sequencing (406×) (Fig. S2A). Two independent PsdR-LasR-MexT mutant lines underwent evolution in the casein-based broth, followed by screening for protease-negative colony mutants using the skim milk agar plate assay (Fig. 1). Notably, one line of these strains had mutant colonies deficient in extracellular protease secretion by day 5 (Fig. S3), whereas another line had such colonies by day 15. These protease-negative phenotypes strongly indicated the acquisition of mutations in the genomes of these mutant colonies. Further evolution of these two strain lines resulted in a “tragedy of the commons” at day 10 and day 18, respectively, as shown by the clarified bacterial fluid, indicating an overload of the parental population by emerging variant mutants deficient in protease production.

**Fig 2:**
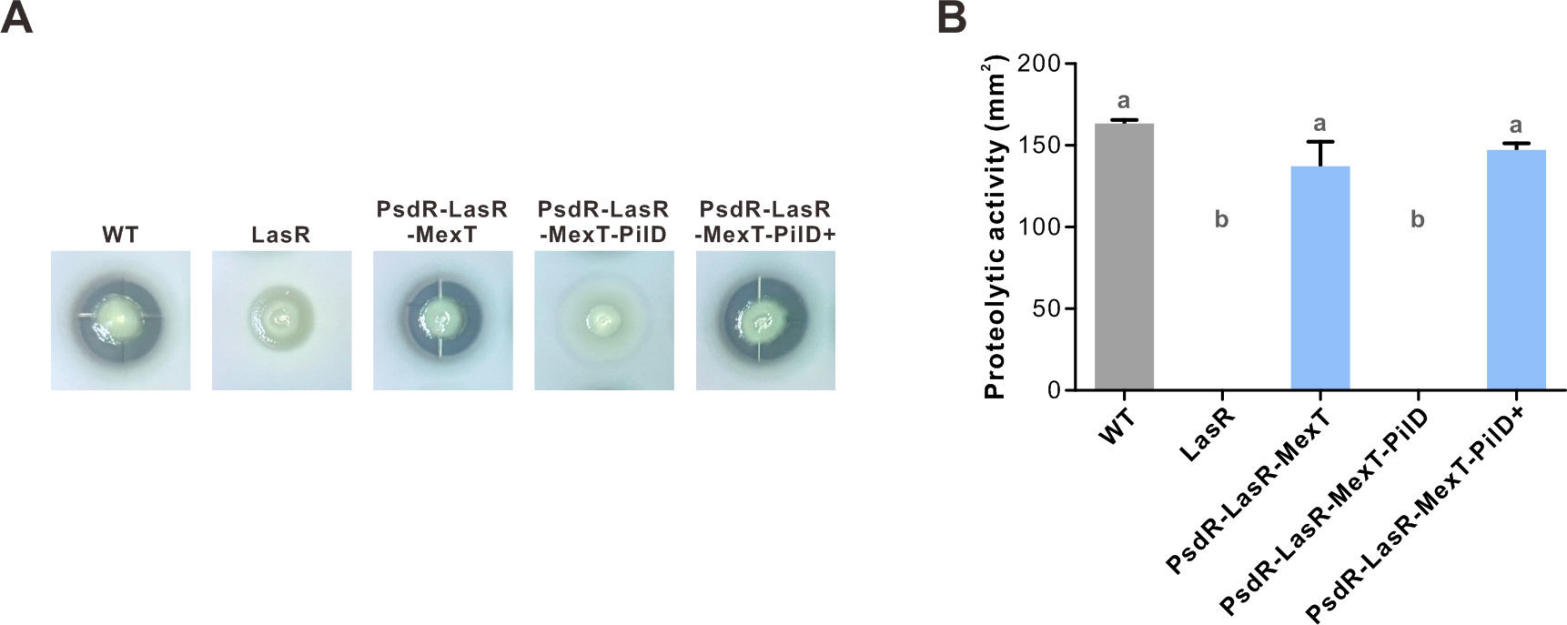
Proteolytic activity of *P. aeruginosa* mutants. (**A**) Proteolytic activity was assessed by skim milk agar plates. Equal amounts of bacteria were spotted onto plates and incubated at 37°C for 24 h, with proteolysis visualized by the zone of clearance. (**B**) Quantitative assessment of proteolytic activity, determined by measuring hydrolysis zone areas with CorelDRAW software (mm²). LasR, LasR-null mutant; PsdR-LasR-MexT, triple deletion mutant; PsdR-LasR-MexT-PilD, *pilD* deletion in PsdR-LasR-MexT; PsdR-LasR-MexT-PilD+, PsdR-LasR-MexT-PilD mutant carrying a single copy of *pilD* driven by a *rrnB* promoter. Data represent mean ± SD (*n* = 4) from three independent experiments. Statistical significance was assessed by one-way ANOVA with Bonferroni post-test. Different letters indicate *P* < 0.05.

**Fig 3:**
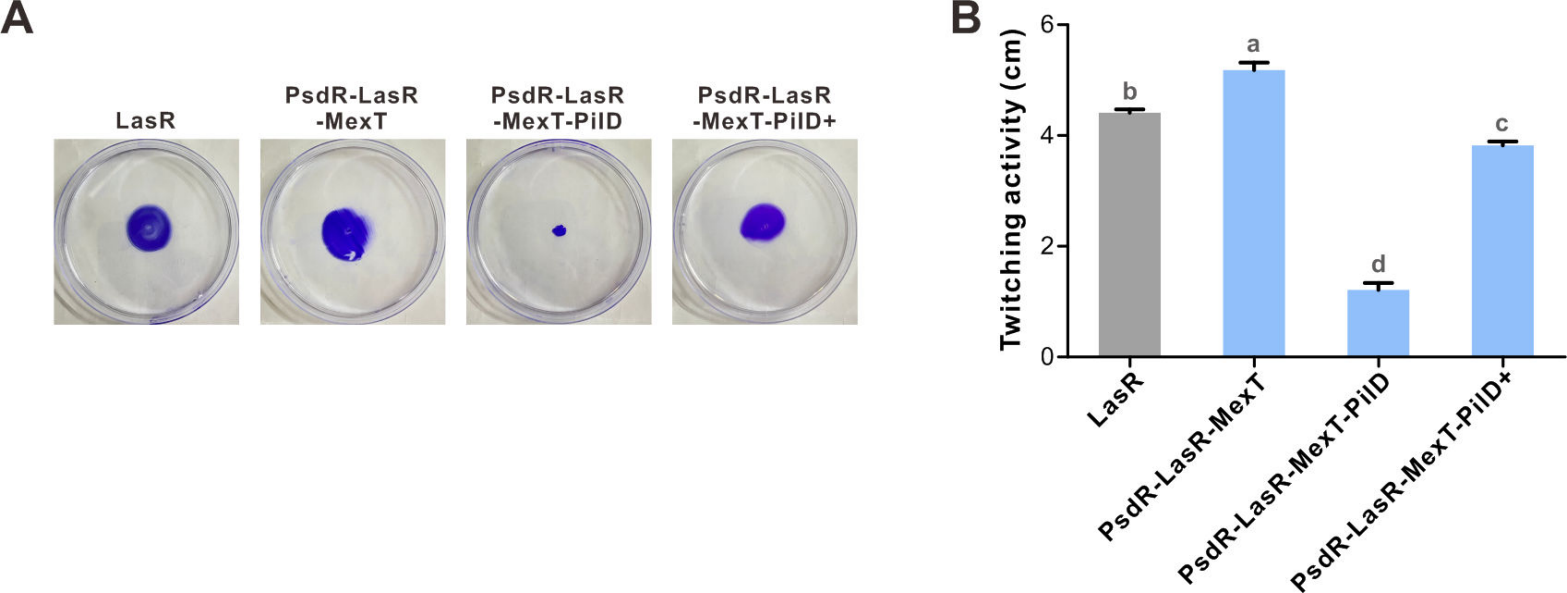
Twitching motility of *P. aeruginosa* mutants. (**A**) Visualization of twitching motility. Equal amounts of indicated bacteria were spotted onto LB plates containing 1% agar and incubated at 37°C for 24 h. Twitching zones were stained with 1% crystal violet and photographed. (**B**) Quantification of twitching motility. The diameter of the twitching zone (cm) was measured using CorelDRAW software. Data are represented as mean ± SD (*n* = 4) from three independent experiments. Statistical significance was determined by one-way ANOVA with Bonferroni post-test analysis. Different letters indicate *P* < 0.05.

To elucidate the underlying mechanisms behind the protease-negative activity, we performed genotyping on the strain line where protease-negative mutant colonies first appeared at day 5. Based on our observations from the preliminary evolution experiment, we specifically examined the *pilD* sequence with PCR amplification and subsequent Sanger sequencing for 10 mutant colonies. The results identified mutated *pilD* (T5073398 → C, Leu235Pro) in these mutant colonies. In addition to the protease-negative phenotype, these mutant colonies were devoid of twitching motility (Fig. S4). Such observed phenotypes of PilD mutants were expected, as PilD is required for pilins processing in the assembly of type IV pilus (54, 55), and a PilD-null mutant is known to exhibit impaired twitching motility. To further affirm the correlation between the observed phenotypic changes and the *pilD* mutations, we generated a PsdR-LasR-MexT-PilD mutant. Similar to the PsdR-LasR-MexT mutant, this constructed mutant was first genome-wide scanned using WGS analysis before formal investigation. No introduced secondary mutations could be identified in this constructed PsdR-LasR-MexT-PilD mutant under our detection conditions (100% genome coverage and 367× sequencing depth) (Fig. S2B). Consistently, this constructed mutant exhibited both protease-negative and twitching-negative phenotypes. Further complementation of this mutant with an episomal copy of wild-type *pilD* successfully restored both proteolytic activity and twitching motility (Fig. 2 and 3). Taken together, our study identified a variant subpopulation containing *pilD* mutations derived from the evolved parental PsdR-LasR-MexT population.

### PilD mutants exhibit cheating behavior when co-cultured with parental MexT mutants

**Fig 4:**
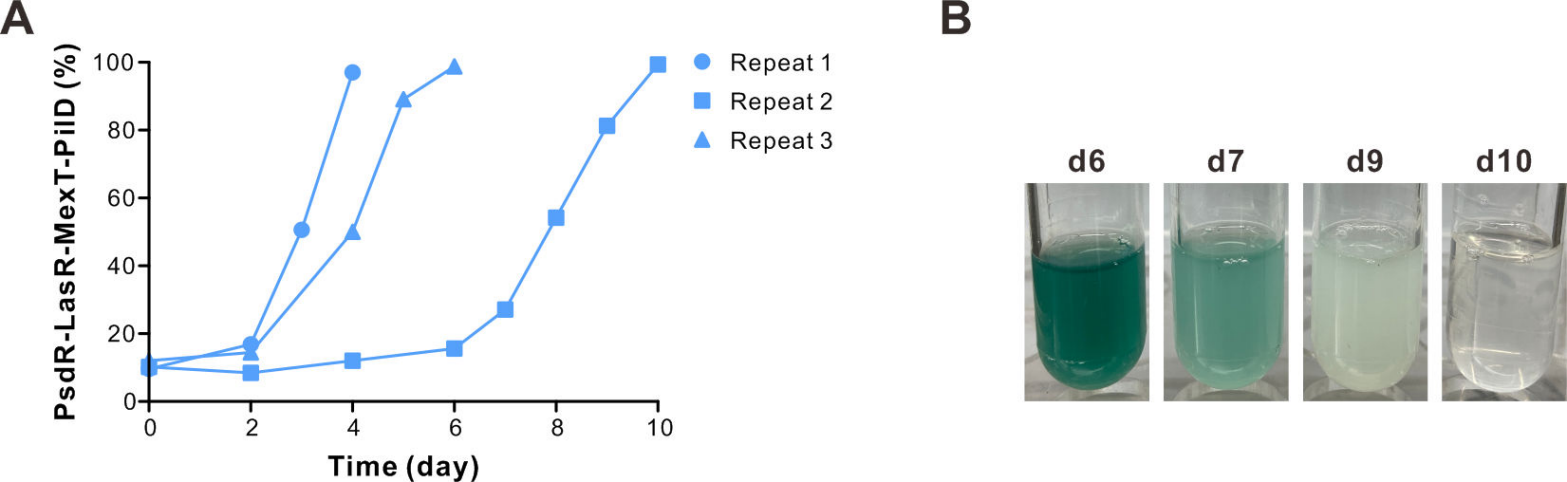
The PsdR-LasR-MexT-PilD mutant outcompetes the parental PsdR-LasR-MexT mutant. (**A**) The PsdR-LasR-MexT mutant was co-cultured with the PsdR-LasR-MexT-PilD mutant at an initial frequency of 90:10 in the casein-based broth. The colonies were counted using the skim milk agar plate assay. (**B**) Photographs of culture tubes at the indicated time. This assay was conducted using three independent strain lines and repeated at least three times with similar results.

To investigate the social role of PilD mutants within QS evolution, we conducted a competition experiment with PsdR-LasR-MexT and PsdR-LasR-MexT-PilD mutants. We first verified that the protease-deficient PsdR-LasR-MexT-PilD mutant exhibited poor growth in the casein-based broth, with its monoculture remained optically clear, closely resembling the blank control even after 48 h of incubation (Fig. S5). Next, the PsdR-LasR-MexT mutant was co-cultured with its derivative PilD mutant at an initial ratio of 90 to 10 in the casein-based broth. The skim milk agar plate assay was employed for distinguishing and quantifying protease-proficient MexT or protease-deficient PilD mutant colonies. Our findings revealed a rapid increase in the percentage of the PilD mutant relative to its parental MexT mutant subpopulation (Fig. 4A). Intriguingly, PilD mutants reached an unexpectedly high population proportion within the population, ultimately leading to a final population collapse across three biologically distinct experiments (Table S2). Concurrently, the population growth, as indicated by the green pigment and turbidity, appeared to decline with the arising PilD mutants within the population (Fig. 4B). Our findings suggest that PilD mutants possess a relative growth advantage over MexT mutants. Our work revealed that the PsdR-LasR-MexT-PilD mutant could outcompete its parental PsdR-LasR-MexT mutant under similar experimental conditions. In summary, the protease-deficient PilD mutants, devoid of bearing the metabolic cost, exhibited cheating behavior by outcompeting their protease-proficient parental MexT mutants, leading to an eventual collapse of the population.

### Inactivation of PilD results in reduced activities of both Rhl and PQS QS circuits

**Fig 5:**
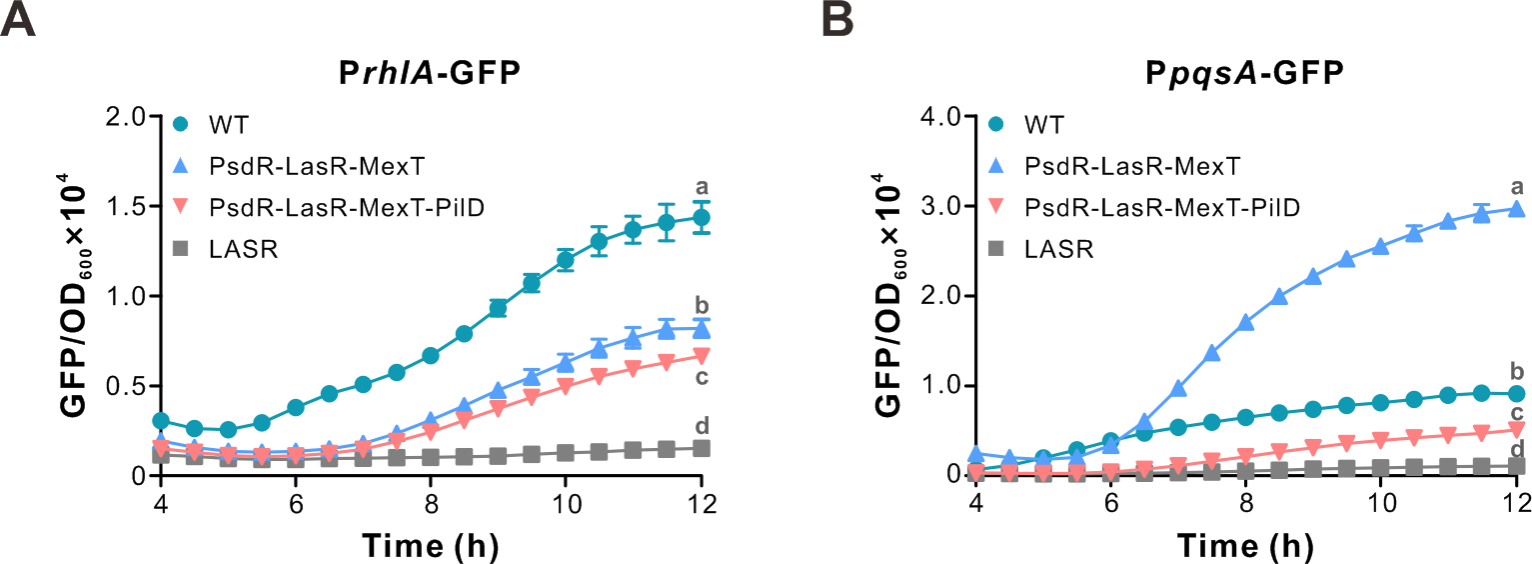
The PsdR-LasR-MexT mutant shows reduced QS-related activities. (**A-B**) Rhl- (**A**) and PQS-responsive (**B**) QS activities of the indicated strains. Rhl- and PQS-responsive QS activities are reflected by the fluorescence levels of the expressed reporters P*rhlA*-GFP and P*pqsA*-GFP, respectively. Fluorescence values, expressed as relative fluorescence units (GFP/OD_600_), were obtained from bacteria cultured in CAA medium for 12 h. Data are presented as means ± SD (*n* = 6). Statistical significance was determined by one-way ANOVA with Bonferroni post-test analysis. Different letters indicate *P* < 0.05. In some cases, the error bars are too small to be seen. The experiment was repeated at least three times with similar results.

To assess the effects of *pilD* deletion on QS activity, we employed two green fluorescence protein (GFP)-based fusion reporter systems, P*rhlA*-GFP and P*pqsA*-GFP, in the study. The P*rhlA*-GFP plasmid contains a core *rhlA* promoter fused with GFP, which reflects the expression level of *rhlA* regulated by RhlR. Similarly, the P*pqsA*-GFP plasmid indicates the expression level of genes in the *pqs* operon. As reported by P*rhlA*-GFP, our result revealed a significant downregulation of the fluorescence signal in the PilD mutant compared with the parental strain (Fig. 5A), indicating reduced Rhl QS activity. An even stronger reduction (5.8-fold at 12 h, *P* < 0.001) of P*pqsA*-GFP expression was observed in the *pilD*-deleting mutant relative to the *pilD*-containing strain (Fig. 5B), suggesting a substantial decrease in PQS QS activity due to the *pilD* deletion.

**Fig 6:**
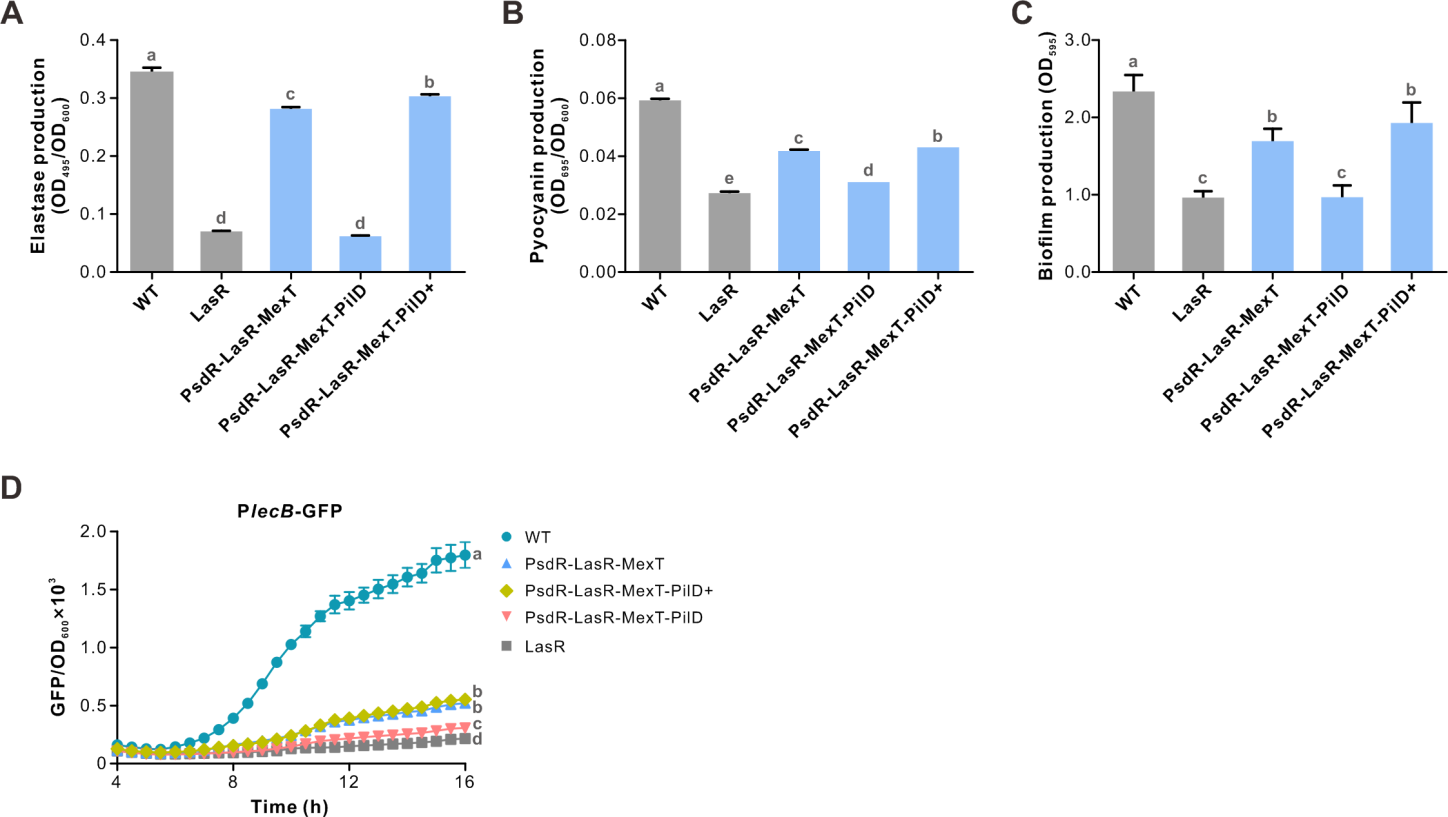
Inactivation of PilD reduces QS-controlled phenotypes. (**A–C**) Relative production of elastase (OD_495_/OD_600_) (**A**), pyocyanin (OD_695_/OD_600_) (**B**), and biofilm (**C**) in the shown strains. Biofilm was visualized by staining with 1% crystal violet. Production of biofilm in the LasR-null mutant was set to 100%. (**D**) *lecB* expression was reduced in the PsdR-LasR-MexT-PilD mutant, as detected by P*lecB*-GFP, a *lecB* promoter-GFP fusion construct. Bacteria carrying P*lecB*-GFP were cultured in CAA medium for 16 h. Data are presented as mean ± SD (*n* ≧ 4) from three independent experiments. Statistical significance was performed by one-way ANOVA with Bonferroni post-test (16 h timepoint in D). Different letters indicate *P* < 0.05.

Next, we examined corresponding phenotypes controlled by QS in the PilD derivatives. As expected, elastase secretion was completely disrupted in the PilD mutant, reducing to the level determined for the LasR-null mutant (Fig. 6A). Consistent with the profile of detected Rhl and PQS QS activities, the production of pyocyanin and biofilm was also decreased in the PilD mutant (Fig. 6B and C). Moreover, using a *lecB* promoter-GFP fusion reporter plasmid (P*lecB*-GFP), we found that the expression level of *lecB* was significantly attenuated in the PilD mutant compared with the control strain (Fig. 6D). The *lecB* gene is known to be responsible for the biosynthesis of the lectin that is primarily regulated by the PQS QS system (56). Taken together, these obtained phenotypic results support the view that the PilD mutant downregulates both Rhl and PQS QS circuits.

### PilD mutants attenuate cytotoxicity on host mammalian cells

**Fig 7:**
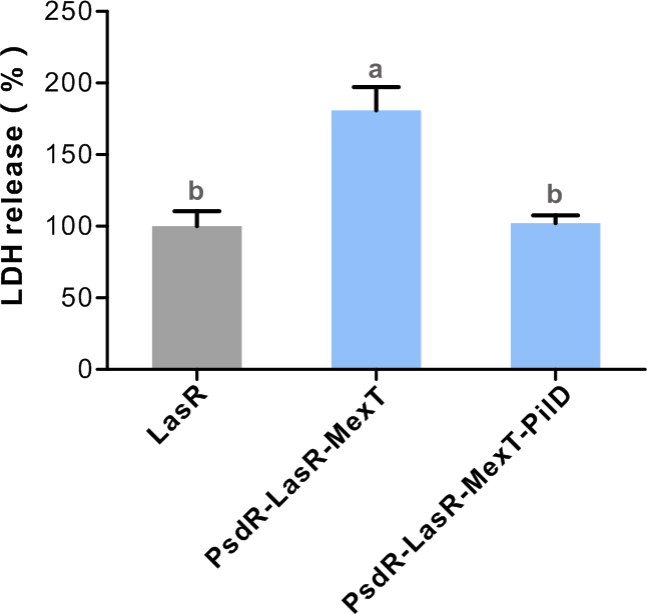
PilD mutants significantly attenuate host cell cytotoxicity. Cytotoxicity levels of *P. aeruginosa* strains were detected by the release of lactate dehydrogenase (LDH) in Chinese hamster ovary (CHO) cells. CHO cells were exposed to equal amounts of the indicated strains for 6 h at a multiplicity of infection (MOI) ≈ 5.0. Data are presented as means ± SD (*n* = 5). Statistical significance was determined by one-way ANOVA with Bonferroni post-test analysis. Different letters indicate *P* < 0.05. The experiment was independently performed three times, and the data obtained were similar.

In this study, we demonstrated that the PilD mutant downregulates both the Rhl and PQS QS circuits and their associated phenotypes. Given that PilD has been reported to control the export of various virulence factors via T2SS or T4P, such as alkaline phosphatase, phospholipase C, and exotoxin A (52), we hypothesized that the inactivation of PilD could impair bacterial pathogenicity. To examine this hypothesis, we assessed the impact of *pilD* expression on bacterial pathogenicity by evaluating its ability to induce cell death in eukaryotic cells. Chinese hamster ovary (CHO) cells were exposed to both the PsdR-LasR-MexT-PilD mutant and control strains, and the extent of host cell death was determined by measuring the released lactate dehydrogenase (LDH). Consistent with previous findings on the restored QS-controlled virulence factors (34, 35), the PsdR-LasR-MexT mutant exhibited significantly higher LDH release in CHO cells when compared with the LasR-null mutant (Fig. 7; Fig. S6). In contrast, a lower level of LDH release was observed in the PsdR-LasR-MexT-PilD mutant relative to its parental MexT mutant. These results demonstrate increased cell death caused by the PsdR-LasR-MexT mutant, whereas decreased cell killing was detected for the PsdR-LasR-MexT-PilD mutant. Based on these findings, we suggest that PilD functions as a virulence regulator, and inactivation of PilD negatively influences bacterial pathogenicity by reducing cytotoxicity toward host mammalian cells.

### Creation of the evolutionary trajectory reveals an iterative social evolution within *P. aeruginosa* population

**Fig 8:**
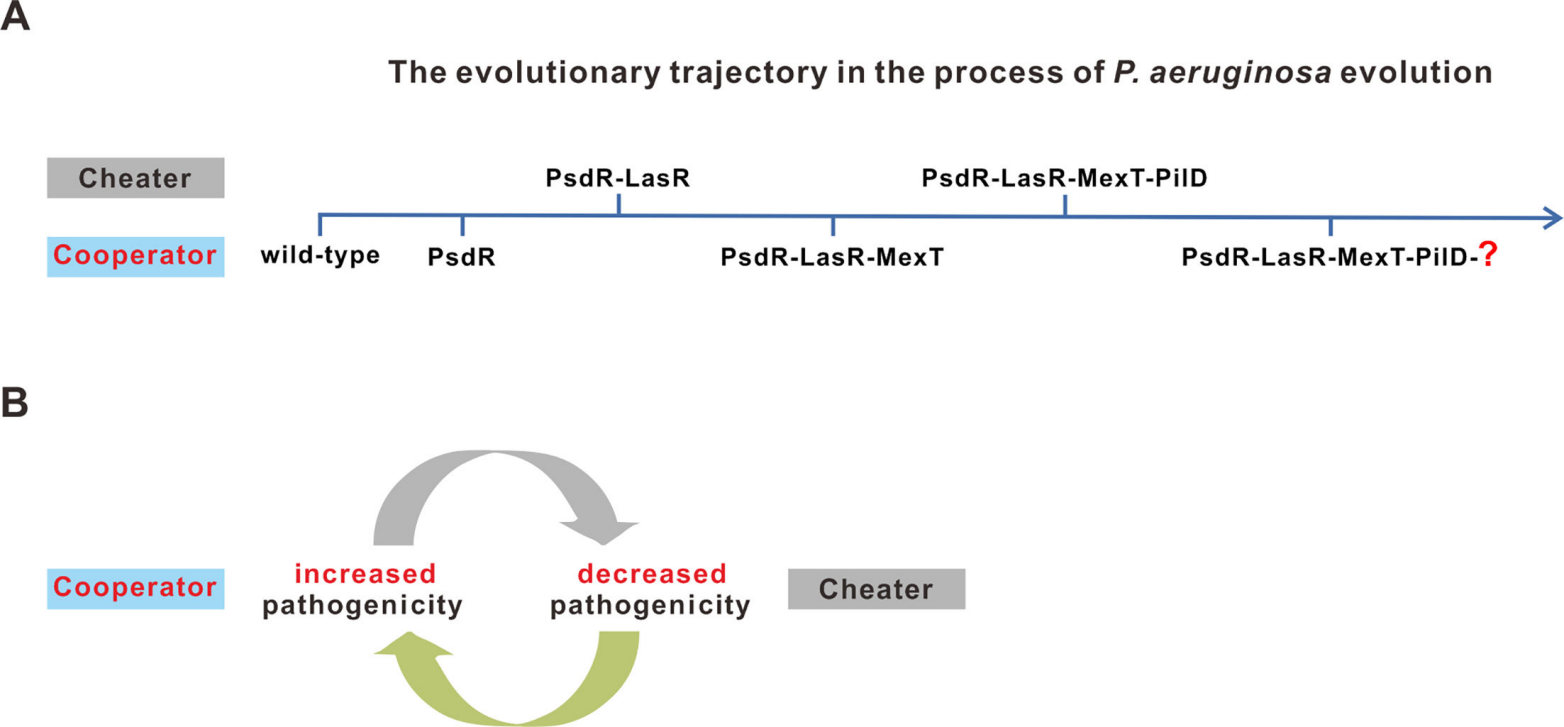
Scheme illustrating the evolutionary trajectory of the *P. aeruginosa* strain PAO1. (**A**) Illustration of the evolutionary trajectory of the *P. aeruginosa* strain PAO1. Variant mutants and their corresponding social roles are shown. (**B**) The iteration of social behaviors between cooperators and cheaters. The changed pathogenicity of the variant mutant is indicated.

Based on previous studies (34–36, 48, 53) and our own experimental results, we summarized the evolutionary trajectory of *P. aeruginosa* within a casein-based broth environment (Fig. 8). In this condition where bacterial QS activation is crucial, variant mutants derived from the wild-type parental population exhibit different QS activities and continue differentiating into distinct QS variants. Mutations in *psdR* quickly appear in the population, conferring improved growth fitness by enhancing dipeptide metabolism (36). Notably, the first emerging QS variant is the LasR mutant, which exhibits QS-inactive activity. Continued evolution of the LasR mutant further reverts it to the MexT mutant, displaying partially restored QS-active phenotypes. Subsequently, the MexT mutant differentiates into the PilD variant, characterized by downregulated QS activities. From a social development perspective, these QS variant mutants engage in an iterative loop, oscillating between cooperators (the wild-type strain and the MexT mutant) and cheaters (the LasR and PilD mutants) throughout the evolutionary process. More importantly, the alteration of social roles among these QS variants also corresponds to changes in variant mutant virulence, underscoring their highly dynamic nature. Taken together, this roadmap of *P. aeruginosa* evolution trajectory establishes a connection between genetic changes and phenotypic consequences and reveals an iterative nature of social behavior in bacterial evolution.

## DISCUSSION

In the present study, we identified a PilD variant, encoding a prepilin peptidase known to participate in both T2SS and T4P (49–51), arising from a population of parental *P. aeruginosa* MexT mutants using an *in vitro* evolution approach. This PilD mutant was characterized as a social cheater due to its deficiency in the secretion of public goods. In previous studies, LasR and PqsR mutants were identified as social cheaters under similar evolution conditions (21–23). These two well-known cheater mutants are both QS signal-blind, downregulating the Las QS and PQS QS systems, respectively. Interestingly, another QS signal-blind RhlR mutant could not be isolated in such evolution experiments, and the underlying mechanisms of this observation remain largely unknown (21). The identification of the PilD cheater mutant in our present study demonstrates that unlike LasR and PqsR mutants, non-QS signal-blind mutants downregulating QS activity are also favored in a QS activation environment. Our findings extend the current understanding of the evolution of cheater mutants. We therefore propose that beyond the currently known typical QS signal-blind mutants, other infection mutants isolated from clinical environments might also be potential social cheaters that have undergone social selection.

In patients with CF chronically infected by *P. aeruginosa*, LasR mutants were frequently isolated (10, 16, 17). These mutants gain a relative growth advantage conferred by social cheating behaviors (22, 23, 26, 57). Interestingly, numerous such LasR mutants retain a QS-active phenotype, producing QS-controlled products, such as pyocyanin and elastase (18, 58, 59). These QS-active phenotypes of LasR mutants have been attributed to the QS revertant, resulting from mutations in *mexT*, a gene encoding the global transcriptional factor (34, 35). These findings suggest that in certain selective environments, LasR social cheaters can revert into cooperators, restoring their cooperative traits. As the cooperator LasR-MexT mutant persists in an environment of selection pressures, we hypothesized that this mutant would continue to evolve, differentiating into unknown variant mutants. In this study, we further investigated the evolutionary trajectory of the LasR-MexT mutant and identified a new variant carrying a mutation in *pilD*, which functions as a social cheater within the cooperative LasR-MexT mutant population. The establishment of the LasR-MexT’s evolutionary trajectory offers insights into the intricate dynamics of social interactions within a bacterial population.

Previous studies have demonstrated that mutations in *mexT* reprogram the hierarchical regulation of QS in LasR-null mutants, thereby transforming LasR-null cheaters into cooperators (34, 35). The continued social evolution under selective pressure highlights the remarkable adaptability of *P. aeruginosa* to changing environments through genomic mutations. This adaptability is consistent with the fact that *P. aeruginosa* contains a complex regulatory network (60). In our current research, we found that further evolution differentiated the LasR-MexT cooperator mutant into a social cheater through acquiring mutations in the *pilD* gene. By establishing the evolutionary trajectory (wild-type strain, LasR, MexT, and PilD mutants), our work reveals a dynamic iteration of social roles between cooperators and cheaters in the process of *P. aeruginosa* evolution. Notably, we observed that the pathogenicity of the corresponding variant mutant also changes in accordance with its social behavior. The discovery of the iterative social behavior of a bacterial subpopulation provides valuable insights into its dynamic nature for the consequent pathogenicity. We hypothesize that similar dynamics of iterative evolution might extend to other clinical pathogen populations under specific selective environments.

It is worth noting that mutations in *pilD* may have evolutionary pleiotropic effects on the mutant population. Since *pilD* codes for a key component of both T2SS and T4P, its inactivation disrupts these two apparatuses, resulting in the abnormal accumulation of a wide range of substrates with diverse functions in the periplasm (61, 62). The disruption of PilD may thus have implications in multiple aspects of bacterial physiology and behavior. In addition, both LasR and MexT are global regulators that affect a variety of cellular functions and pathways beyond QS regulation (32, 63). LasR mutants, for instance, were found to enhance growth on specific carbon and nitrogen sources, indicating a broader metabolic adaptation (16). Additionally, LasR mutants confer a fitness advantage in microoxic conditions, suggesting their role in environmental adaptation (57). Similarly, MexT governs the expression of operon genes involved in detoxification, nutrient-scavenging, and bacteria-host interaction, highlighting its association with multiple cellular processes (32). Therefore, the combined deletion of these three global regulators in the mutant population would have profound impacts on bacterial physiology and behavior. Understanding the comprehensive consequences of these pleiotropic effects would shed light on the complex interplay among regulatory pathways and their impacts on bacterial fitness.

*P. aeruginosa* strains infecting the lungs of cystic fibrosis patients have been described as being localized to a small region within the lung, creating a localized competitive environment for the bacterial population (64). QS mutants of *P. aeruginosa* isolated from clinical infections are believed to arise as a result of social consequences related to their growth fitness, as they exploit extracellular products from parental populations or other microorganisms. For example, LasR mutants were frequently isolated from acute and chronic infections (10, 16, 17). The emergence of LasR mutants in clinical environments was attributed to their social cheating behavior, as demonstrated in evolutionary experiments and a mouse infection model (22, 23, 26). Based on the identification and characterization of PilD mutants in our current study, we propose that similar to LasR mutants, PilD mutants might also function as social cheaters, gaining a growth advantage within natural populations under clinical conditions. In fact, our preliminary bioinformatic analysis supports this hypothesis by revealing the prevalence of PilD mutants among clinical *P. aeruginosa* isolates (accompanying manuscript).

Our findings revealed that PQS QS activity was substantially reduced in the PilD mutant. Previous findings have demonstrated that the Las QS system positively regulates the PQS QS circuit (65), whereas the Rhl QS system negatively controls the expression of *pqsR* and *pqsABCDE* operon genes (66). Notably, we observed only a slight decrease in RhlR-responsive activity (P*rhlA*-GFP) in the PilD mutant. Therefore, we speculated that the down-regulation of PQS QS activity is not attributed to the Rhl QS system but rather involves T2SS- or T4P-associated regulatory mechanisms. Since PilD acts as a prepilin peptidase involved in T2SS and T4P (49–51), it is very unlikely that PilD directly modulates the PQS QS circuit. Indeed, neither the PQS signal receptor PqsR nor the enzymes essential for the biosynthesis of PQS or its precursor HHQ have been reported to be transported via T2SS or T4P (51, 67). Considering that T2SS and T4P associate with a broad spectrum of substrates with diverse functions (61, 62), we suggest that PilD inactivation might indirectly influence the PQS QS circuit through proteins trapped in the periplasm when T2SS or T4P is disrupted.

## Data Availability

The data sets generated during the current study have been deposited in the NCBI SRA database (https://www.ncbi.nlm.nih.gov/sra) under the accession number PRJNA1304192.

## SUPPLEMENTAL MATERIAL

Supplemental figures aem.00915-25-s0001.docx Figures S1 to S6.

Table S1 aem.00915-25-s0002.xlsx Identification of mutations in evolved colonies from the LasR-null strain.

Table S2 aem.00915-25-s0003.xlsx Percentage of PsdR-LasR-MexT-PilD mutants in competition experiment.

Table S3 aem.00915-25-s0004.xlsx Strains and plasmids used in this study.

Table S4 aem.00915-25-s0005.xlsx Oligonucleotides used in this study.

ASM does not own the copyrights to Supplemental Material that may be linked to, or accessed through, an article. The authors have granted ASM a non-exclusive, world-wide license to publish the Supplemental Material files. Please contact the corresponding author directly for reuse.

## References

[1] Gellatly SL, Hancock REW. 2013. Pseudomonas aeruginosa: new insights into pathogenesis and host defenses. Pathog Dis 67:159–173. doi:10.1111/2049-632X.12033 23620179

[2] Klockgether J, Tümmler B. 2017. Recent advances in understanding Pseudomonas aeruginosa as a pathogen. F1000Res 6:1261. doi:10.12688/f1000research.10506.1 28794863 PMC5538032

[3] Papenfort K, Bassler BL. 2016. Quorum sensing signal-response systems in Gram-negative bacteria. Nat Rev Microbiol 14:576–588. doi:10.1038/nrmicro.2016.89 27510864 PMC5056591

[4] Azimi S, Klementiev AD, Whiteley M, Diggle SP. 2020. Bacterial quorum sensing during infection. Annu Rev Microbiol 74:201–219. doi:10.1146/annurev-micro-032020-093845 32660382 PMC13064819

[5] Andersson DI, Hughes D. 2014. Microbiological effects of sublethal levels of antibiotics. Nat Rev Microbiol 12:465–478. doi:10.1038/nrmicro3270 24861036

[6] Fodor AA, Klem ER, Gilpin DF, Elborn JS, Boucher RC, Tunney MM, Wolfgang MC. 2012. The adult cystic fibrosis airway microbiota is stable over time and infection type, and highly resilient to antibiotic treatment of exacerbations. PLoS One 7:e45001. doi:10.1371/journal.pone.0045001 23049765 PMC3458854

[7] Lopes SP, Azevedo NF, Pereira MO. 2015. Microbiome in cystic fibrosis: shaping polymicrobial interactions for advances in antibiotic therapy. Crit Rev Microbiol 41:353–365. doi:10.3109/1040841X.2013.847898 24645634

[8] Winstanley C, O’Brien S, Brockhurst MA. 2016. Pseudomonas aeruginosa evolutionary adaptation and diversification in cystic fibrosis chronic lung infections. Trends Microbiol 24:327–337. doi:10.1016/j.tim.2016.01.008 26946977 PMC4854172

[9] Rossi E, La Rosa R, Bartell JA, Marvig RL, Haagensen JAJ, Sommer LM, Molin S, Johansen HK. 2021. Pseudomonas aeruginosa adaptation and evolution in patients with cystic fibrosis. Nat Rev Microbiol 19:331–342. doi:10.1038/s41579-020-00477-5 33214718

[10] Smith EE, Buckley DG, Wu Z, Saenphimmachak C, Hoffman LR, D’Argenio DA, Miller SI, Ramsey BW, Speert DP, Moskowitz SM, Burns JL, Kaul R, Olson MV. 2006. Genetic adaptation by Pseudomonas aeruginosa to the airways of cystic fibrosis patients. Proc Natl Acad Sci USA 103:8487–8492. doi:10.1073/pnas.0602138103 16687478 PMC1482519

[11] Yang L, Jelsbak L, Marvig RL, Damkiær S, Workman CT, Rau MH, Hansen SK, Folkesson A, Johansen HK, Ciofu O, Høiby N, Sommer MOA, Molin S. 2011. Evolutionary dynamics of bacteria in a human host environment. Proc Natl Acad Sci USA 108:7481–7486. doi:10.1073/pnas.1018249108 21518885 PMC3088582

[12] Bragonzi A, Paroni M, Nonis A, Cramer N, Montanari S, Rejman J, Di Serio C, Döring G, Tümmler B. 2009. Pseudomonas aeruginosa microevolution during cystic fibrosis lung infection establishes clones with adapted virulence. Am J Respir Crit Care Med 180:138–145. doi:10.1164/rccm.200812-1943OC 19423715

[13] Cramer N, Klockgether J, Wrasman K, Schmidt M, Davenport CF, Tümmler B. 2011. Microevolution of the major common Pseudomonas aeruginosa clones C and PA14 in cystic fibrosis lungs. Environ Microbiol 13:1690–1704. doi:10.1111/j.1462-2920.2011.02483.x 21492363

[14] Hoboth C, Hoffmann R, Eichner A, Henke C, Schmoldt S, Imhof A, Heesemann J, Hogardt M. 2009. Dynamics of adaptive microevolution of hypermutable Pseudomonas aeruginosa during chronic pulmonary infection in patients with cystic fibrosis. J Infect Dis 200:118–130. doi:10.1086/599360 19459782

[15] Marvig RL, Dolce D, Sommer LM, Petersen B, Ciofu O, Campana S, Molin S, Taccetti G, Johansen HK. 2015. Within-host microevolution of Pseudomonas aeruginosa in Italian cystic fibrosis patients. BMC Microbiol 15:218. doi:10.1186/s12866-015-0563-9 26482905 PMC4612410

[16] D’Argenio DA, Wu M, Hoffman LR, Kulasekara HD, Déziel E, Smith EE, Nguyen H, Ernst RK, Larson Freeman TJ, Spencer DH, Brittnacher M, Hayden HS, Selgrade S, Klausen M, Goodlett DR, Burns JL, Ramsey BW, Miller SI. 2007. Growth phenotypes of Pseudomonas aeruginosa lasR mutants adapted to the airways of cystic fibrosis patients. Mol Microbiol 64:512–533. doi:10.1111/j.1365-2958.2007.05678.x 17493132 PMC2742308

[17] Köhler T, Buckling A, van Delden C. 2009. Cooperation and virulence of clinical Pseudomonas aeruginosa populations. Proc Natl Acad Sci USA 106:6339–6344. doi:10.1073/pnas.0811741106 19332772 PMC2669332

[18] Feltner JB, Wolter DJ, Pope CE, Groleau M-C, Smalley NE, Greenberg EP, Mayer-Hamblett N, Burns J, Déziel E, Hoffman LR, Dandekar AA. 2016. LasR variant cystic fibrosis isolates reveal an adaptable quorum-sensing hierarchy in Pseudomonas aeruginosa. mBio 7:e01513-16. doi:10.1128/mBio.01513-16 27703072 PMC5050340

[19] Andersen SB, Marvig RL, Molin S, Krogh Johansen H, Griffin AS. 2015. Long-term social dynamics drive loss of function in pathogenic bacteria. Proc Natl Acad Sci USA 112:10756–10761. doi:10.1073/pnas.1508324112 26240352 PMC4553784

[20] Jayakumar P, Figueiredo ART, Kümmerli R. 2022. Evolution of quorum sensing in Pseudomonas aeruginosa can occur via loss of function and regulon modulation. mSystems 7:e0035422. doi:10.1128/msystems.00354-22 36190124 PMC9600717

[21] Wilder CN, Diggle SP, Schuster M. 2011. Cooperation and cheating in Pseudomonas aeruginosa: the roles of the las, rhl and pqs quorum-sensing systems. ISME J 5:1332–1343. doi:10.1038/ismej.2011.13 21368905 PMC3146268

[22] Sandoz KM, Mitzimberg SM, Schuster M. 2007. Social cheating in Pseudomonas aeruginosa quorum sensing. Proc Natl Acad Sci USA 104:15876–15881. doi:10.1073/pnas.0705653104 17898171 PMC2000394

[23] Diggle SP, Griffin AS, Campbell GS, West SA. 2007. Cooperation and conflict in quorum-sensing bacterial populations. Nature 450:411–414. doi:10.1038/nature06279 18004383

[24] Gurney J, Azimi S, Brown SP, Diggle SP. 2020. Combinatorial quorum sensing in Pseudomonas aeruginosa allows for novel cheating strategies. Microbiology (Reading) 166:777–784. doi:10.1099/mic.0.000941 32511085

[25] Scott-Phillips TC, Gurney J, Ivens A, Diggle SP, Popat R. 2014. Combinatorial communication in bacteria: implications for the origins of linguistic generativity. PLoS One 9:e95929. doi:10.1371/journal.pone.0095929 24759740 PMC3997515

[26] Rumbaugh KP, Diggle SP, Watters CM, Ross-Gillespie A, Griffin AS, West SA. 2009. Quorum sensing and the social evolution of bacterial virulence. Curr Biol 19:341–345. doi:10.1016/j.cub.2009.01.050 19230668

[27] Olivas AD, Shogan BD, Valuckaite V, Zaborin A, Belogortseva N, Musch M, Meyer F, Trimble WL, An G, Gilbert J, Zaborina O, Alverdy JC. 2012. Intestinal tissues induce an SNP mutation in Pseudomonas aeruginosa that enhances its virulence: possible role in anastomotic leak. PLoS One 7:e44326. doi:10.1371/journal.pone.0044326 22952955 PMC3432121

[28] Luong PM, Shogan BD, Zaborin A, Belogortseva N, Shrout JD, Zaborina O, Alverdy JC. 2014. Emergence of the P2 phenotype in Pseudomonas aeruginosa PAO1 strains involves various mutations in mexT or mexF. J Bacteriol 196:504–513. doi:10.1128/JB.01050-13 24244000 PMC3911258

[29] Maseda H, Saito K, Nakajima A, Nakae T. 2000. Variation of the mexT gene, a regulator of the MexEF-oprN efflux pump expression in wild-type strains of Pseudomonas aeruginosa. FEMS Microbiol Lett 192:107–112. doi:10.1111/j.1574-6968.2000.tb09367.x 11040437

[30] Köhler T, Epp SF, Curty LK, Pechère JC. 1999. Characterization of MexT, the regulator of the MexE-MexF-OprN multidrug efflux system of Pseudomonas aeruginosa. J Bacteriol 181:6300–6305. doi:10.1128/JB.181.20.6300-6305.1999 10515918 PMC103763

[31] Maddocks SE, Oyston PCF. 2008. Structure and function of the LysR-type transcriptional regulator (LTTR) family proteins. Microbiology (Reading) 154:3609–3623. doi:10.1099/mic.0.2008/022772-0 19047729

[32] Tian Z-X, Fargier E, Mac Aogáin M, Adams C, Wang Y-P, O’Gara F. 2009. Transcriptome profiling defines a novel regulon modulated by the LysR-type transcriptional regulator MexT in Pseudomonas aeruginosa. Nucleic Acids Res 37:7546–7559. doi:10.1093/nar/gkp828 19846594 PMC2794183

[33] Lamarche MG, Déziel E. 2011. MexEF-OprN efflux pump exports the Pseudomonas quinolone signal (PQS) precursor HHQ (4-hydroxy-2-heptylquinoline). PLoS One 6:e24310. doi:10.1371/journal.pone.0024310 21957445 PMC3177830

[34] Oshri RD, Zrihen KS, Shner I, Omer Bendori S, Eldar A. 2018. Selection for increased quorum-sensing cooperation in Pseudomonas aeruginosa through the shut-down of a drug resistance pump. ISME J 12:2458–2469. doi:10.1038/s41396-018-0205-y 29925881 PMC6154968

[35] Kostylev M, Kim DY, Smalley NE, Salukhe I, Greenberg EP, Dandekar AA. 2019. Evolution of the Pseudomonas aeruginosa quorum-sensing hierarchy. Proc Natl Acad Sci USA 116:7027–7032. doi:10.1073/pnas.1819796116 30850547 PMC6452656

[36] Asfahl KL, Walsh J, Gilbert K, Schuster M. 2015. Non-social adaptation defers a tragedy of the commons in Pseudomonas aeruginosa quorum sensing. ISME J 9:1734–1746. doi:10.1038/ismej.2014.259 25615439 PMC4511930

[37] Rietsch A, Vallet-Gely I, Dove SL, Mekalanos JJ. 2005. ExsE, a secreted regulator of type III secretion genes in Pseudomonas aeruginosa. Proc Natl Acad Sci USA 102:8006–8011. doi:10.1073/pnas.0503005102 15911752 PMC1142391

[38] Murray HD, Gourse RL. 2004. Unique roles of the rrn P2 rRNA promoters in Escherichia coli. Mol Microbiol 52:1375–1387. doi:10.1111/j.1365-2958.2004.04060.x 15165240

[39] Choi K-H, Schweizer HP. 2006. Mini-Tn7 insertion in bacteria with single attTn7 sites: example Pseudomonas aeruginosa. Nat Protoc 1:153–161. doi:10.1038/nprot.2006.24 17406227

[40] Li H, Durbin R. 2009. Fast and accurate short read alignment with Burrows-Wheeler transform. Bioinformatics 25:1754–1760. doi:10.1093/bioinformatics/btp324 19451168 PMC2705234

[41] Martin M. 2011. Cutadapt removes adapter sequences from high-throughput sequencing reads. EMBnet j 17:10. doi:10.14806/ej.17.1.200

[42] Li H, Handsaker B, Wysoker A, Fennell T, Ruan J, Homer N, Marth G, Abecasis G, Durbin R, 1000 Genome Project Data Processing Subgroup. 2009. The sequence Alignment/Map format and SAMtools. Bioinformatics 25:2078–2079. doi:10.1093/bioinformatics/btp352 19505943 PMC2723002

[43] Kim D, Langmead B, Salzberg SL. 2015. HISAT: a fast spliced aligner with low memory requirements. Nat Methods 12:357–360. doi:10.1038/nmeth.3317 25751142 PMC4655817

[44] Anders S, Pyl PT, Huber W. 2015. HTSeq—a Python framework to work with high-throughput sequencing data. Bioinformatics 31:166–169. doi:10.1093/bioinformatics/btu638 25260700 PMC4287950

[45] Love MI, Huber W, Anders S. 2014. Moderated estimation of fold change and dispersion for RNA-seq data with DESeq2. Genome Biol 15:550. doi:10.1186/s13059-014-0550-8 25516281 PMC4302049

[46] Kiely PD, O’Callaghan J, Abbas A, O’Gara F. 2008. Genetic analysis of genes involved in dipeptide metabolism and cytotoxicity in Pseudomonas aeruginosa PAO1. Microbiology (Reading) 154:2209–2218. doi:10.1099/mic.0.2007/015032-0 18667554

[47] Trouillon J, Ragno M, Simon V, Attrée I, Elsen S. 2021. Transcription inhibitors with XRE DNA-binding and cupin signal-sensing domains drive metabolic diversification in Pseudomonas. mSystems 6:e00753-20. doi:10.1128/mSystems.00753-20 33436508 PMC7901475

[48] Qiu H, Li Y, Yuan M, Chen H, Dandekar AA, Dai W. 2024. Uncovering a hidden functional role of the XRE-cupin protein PsdR as a novel quorum-sensing regulator in Pseudomonas aeruginosa. PLoS Pathog 20:e1012078. doi:10.1371/journal.ppat.1012078 38484003 PMC10965056

[49] Bally M, Filloux A, Akrim M, Ball G, Lazdunski A, Tommassen J. 1992. Protein secretion in Pseudomonas aeruginosa: characterization of seven xcp genes and processing of secretory apparatus components by prepilin peptidase. Mol Microbiol 6:1121–1131. doi:10.1111/j.1365-2958.1992.tb01550.x 1588814

[50] Nunn DN, Lory S. 1992. Components of the protein-excretion apparatus of Pseudomonas aeruginosa are processed by the type IV prepilin peptidase. Proc Natl Acad Sci USA 89:47–51. doi:10.1073/pnas.89.1.47 1309616 PMC48172

[51] Filloux A. 2011. Protein secretion systems in Pseudomonas aeruginosa: an essay on diversity, evolution, and function. Front Microbiol 2:155. doi:10.3389/fmicb.2011.00155 21811488 PMC3140646

[52] Strom MS, Nunn D, Lory S. 1991. Multiple roles of the pilus biogenesis protein pilD: involvement of pilD in excretion of enzymes from Pseudomonas aeruginosa. J Bacteriol 173:1175–1180. doi:10.1128/jb.173.3.1175-1180.1991 1671384 PMC207239

[53] Jiang B, Qiu H, Lu C, Lu M, Li Y, Dai W. 2024. Uncovering the GacS-mediated role in evolutionary progression through trajectory reconstruction in Pseudomonas aeruginosa. Nucleic Acids Res 52:3856–3869. doi:10.1093/nar/gkae187 38477346 PMC11040156

[54] Giltner CL, Habash M, Burrows LL. 2010. Pseudomonas aeruginosa minor pilins are incorporated into type IV pili. J Mol Biol 398:444–461. doi:10.1016/j.jmb.2010.03.028 20338182

[55] Strom MS, Lory S. 1991. Amino acid substitutions in pilin of Pseudomonas aeruginosa. Effect on leader peptide cleavage, amino-terminal methylation, and pilus assembly. J Biol Chem 266:1656–1664. 1671038

[56] Lin J, Cheng J, Wang Y, Shen X. 2018. The Pseudomonas quinolone signal (PQS): not just for quorum sensing anymore. Front Cell Infect Microbiol 8:230. doi:10.3389/fcimb.2018.00230 30023354 PMC6039570

[57] Clay ME, Hammond JH, Zhong F, Chen X, Kowalski CH, Lee AJ, Porter MS, Hampton TH, Greene CS, Pletneva EV, Hogan DA. 2020. Pseudomonas aeruginosa lasR mutant fitness in microoxia is supported by an Anr-regulated oxygen-binding hemerythrin. Proc Natl Acad Sci USA 117:3167–3173. doi:10.1073/pnas.1917576117 31980538 PMC7022198

[58] Cruz RL, Asfahl KL, Van den Bossche S, Coenye T, Crabbé A, Dandekar AA. 2020. RhlR-regulated acyl-homoserine lactone quorum sensing in a cystic fibrosis isolate of Pseudomonas aeruginosa. mBio 11:e00532-20. doi:10.1128/mBio.00532-20 32265330 PMC7157775

[59] Bjarnsholt T, Jensen PØ, Jakobsen TH, Phipps R, Nielsen AK, Rybtke MT, Tolker-Nielsen T, Givskov M, Høiby N, Ciofu O, Scandinavian Cystic Fibrosis Study Consortium. 2010. Quorum sensing and virulence of Pseudomonas aeruginosa during lung infection of cystic fibrosis patients. PLoS One 5:e10115. doi:10.1371/journal.pone.0010115 20404933 PMC2853559

[60] Huang H, Shao X, Xie Y, Wang T, Zhang Y, Wang X, Deng X. 2019. An integrated genomic regulatory network of virulence-related transcriptional factors in Pseudomonas aeruginosa. Nat Commun 10:1–13. doi:10.1038/s41467-019-10778-w 31270321 PMC6610081

[61] Green ER, Mecsas J. 2016. Bacterial secretion systems: an overview. Microbiol Spectr 4. doi:10.1128/microbiolspec.VMBF-0012-2015 PMC4804464 26999395

[62] Nivaskumar M, Francetic O. 2014. Type II secretion system: a magic beanstalk or a protein escalator. Biochim Biophys Acta 1843:1568–1577. doi:10.1016/j.bbamcr.2013.12.020 24389250

[63] Gilbert KB, Kim TH, Gupta R, Greenberg EP, Schuster M. 2009. Global position analysis of the Pseudomonas aeruginosa quorum-sensing transcription factor LasR. Mol Microbiol 73:1072–1085. doi:10.1111/j.1365-2958.2009.06832.x 19682264 PMC2759405

[64] Jones AM, Govan JRW, Doherty CJ, Dodd ME, Isalska BJ, Stanbridge TN, Webb AK. 2003. Identification of airborne dissemination of epidemic multiresistant strains of Pseudomonas aeruginosa at a CF centre during a cross infection outbreak. Thorax 58:525–527. doi:10.1136/thorax.58.6.525 12775867 PMC1746694

[65] Schertzer JW, Boulette ML, Whiteley M. 2009. More than a signal: non-signaling properties of quorum sensing molecules. Trends Microbiol 17:189–195. doi:10.1016/j.tim.2009.02.001 19375323

[66] Cao H, Krishnan G, Goumnerov B, Tsongalis J, Tompkins R, Rahme LG. 2001. A quorum sensing-associated virulence gene of Pseudomonas aeruginosa encodes a LysR-like transcription regulator with a unique self-regulatory mechanism. Proc Natl Acad Sci USA 98:14613–14618. doi:10.1073/pnas.251465298 11724939 PMC64730

[67] Pena RT, Blasco L, Ambroa A, González-Pedrajo B, Fernández-García L, López M, Bleriot I, Bou G, García-Contreras R, Wood TK, Tomás M. 2019. Relationship between quorum sensing and secretion systems. Front Microbiol 10:1100. doi:10.3389/fmicb.2019.01100 31231316 PMC6567927

