# Supplementary material for: Identification of PilD mutants reveals the iterative social evolution in Pseudomonas aeruginosa
Source: Appl Environ Microbiol. 2025 Sep 4;91(10):e00915-25. doi: 10.1128/aem.00915-25 (PMC12542647; doi:10.1128/aem.00915-25)
Supplement: Supplemental figures — Figures S1 to S6. [file aem.00915-25-s0001.docx]

**Supplementary Information**

Identification of PilD mutants reveals the iterative social evolution in *Pseudomonas aeruginosa*

Huifang Qiu^1^, Xiaoqing Zhou^1^, Weijun Dai^1*^

**Supplementary figures**

**Figure S1.** Screen for mutant colonies of the LasR-null strain.

**Figure S2.** Whole-genome sequencing (WGS) analysis of two constructed mutants.

**Figure S3.** Identification of protease-negative colony mutants of the PsdR-LasR-MexT population.

**Figure S4.** Twitching motility of mutants isolated from the parental PsdR-LasR-MexT population.

**Figure S5.** Growth phenotypes of *P. aeruginosa* strains in casein-based broth.

**Figure S6.** Cell cytotoxicity of assayed strains.

**Supplementary tables**

**Table S1.** Identification of mutations in evolved colonies from the LasR-null strain.

**Table S2.** Percentage of PsdR-LasR-MexT-PilD mutant in competition experiment.

**Table S3.** Strains and plasmids used in this study.

**Table S4.** Oligonucleotides used in this study.


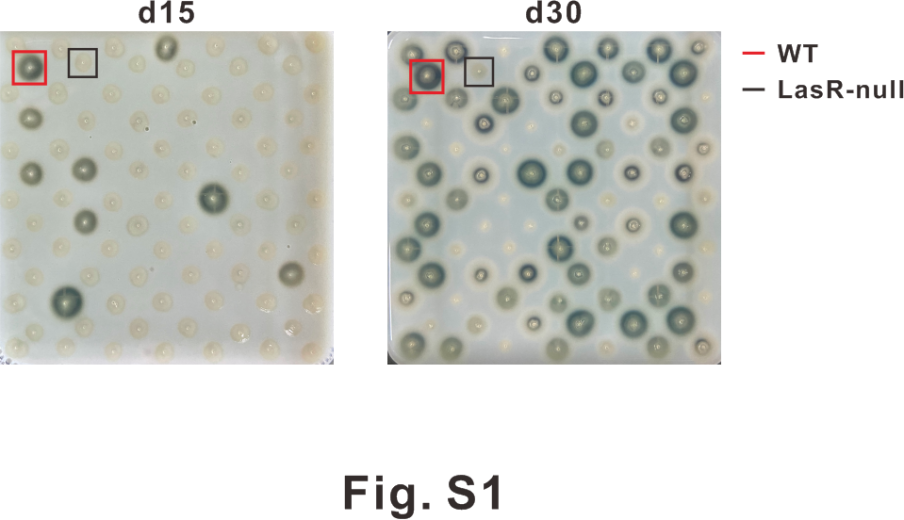


**Figure S1. Screen for mutant colonies of the LasR-null strain.** Mutant colonies evolved from the LasR-null strain were screened based on their protease phenotypes. Bacterial colonies were examined for extracellular protease activity at different time points using skim milk agar plates (control strains: WT, wild-type strain PAO1; LasR, LasR-null mutant). Red rectangle shows the WT strain. Black rectangle indicates the LasR-null strain.


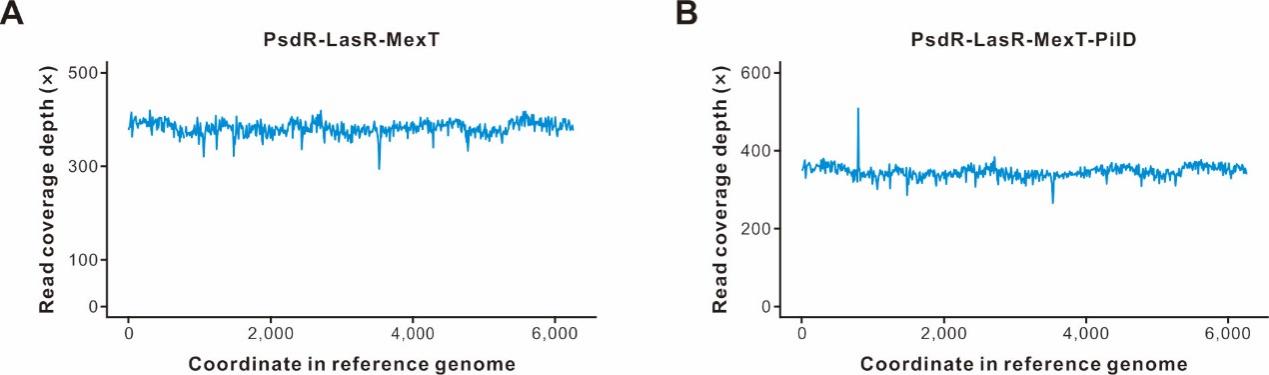


**Figure S2. Whole-genome sequencing (WGS) analysis of two constructed mutants.** Read coverage depth of whole genome sequencing of the PsdR-LasR-MexT mutant (A) and the PsdR-LasR-MexT-PilD mutant (B). Average fold-coverages within a 10 kb window are plotted against the *P. aeruginosa* PAO1 reference genome.


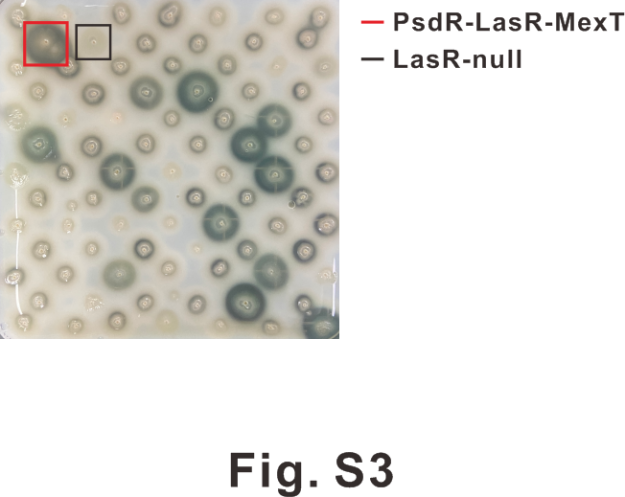


**Figure S3. Identification of protease-negative colony mutants of the PsdR-LasR-MexT population.** The PsdR-LasR-MexT population was serially transferred in casein-based broth and screened for protease-negative mutants using a skim milk agar plate assay. The black rectangle indicates protease-negative control strain, the LasR-null mutant. The red rectangle shows protease-positive control strain, the parental PsdR-LasR-MexT mutant.


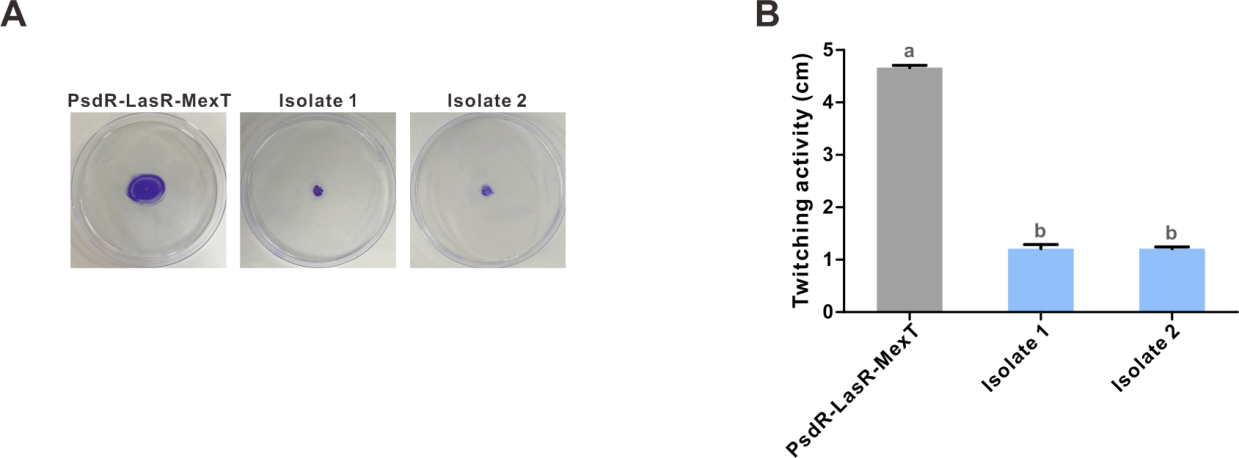


**Figure S4. Twitching motility of mutants isolated from the parental PsdR-LasR-MexT population.** (A) Visualization and (B) Quantitative of twitching motility. Bacterial twitching motility was assayed using LB agar plate (1.0%). The diameter of twitching zone was visualized by 1% crystal violet staining and measured using CorelDRAW software. PsdR-LasR-MexT, triple deletion mutant; Isolate 1/2, obtained protease-negative mutants. Data are represented as mean ± SD (*n* = 4) from three independent experiments. Statistical significance was determined by one-way ANOVA with Bonferroni post test analysis. Different letters indicate *P* < 0.05.


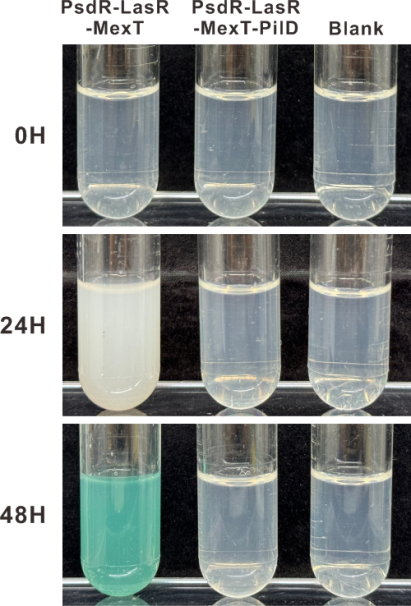


**Figure S5. Growth phenotypes of *P. aeruginosa* strains in casein-based broth.** The PsdR-LasR-MexT mutant and PsdR-LasR-MexT-PilD mutant were monocultured in casein-based broth, with sterile broth serving as negative control (Blank). Photographs were taken at 24 h intervals.


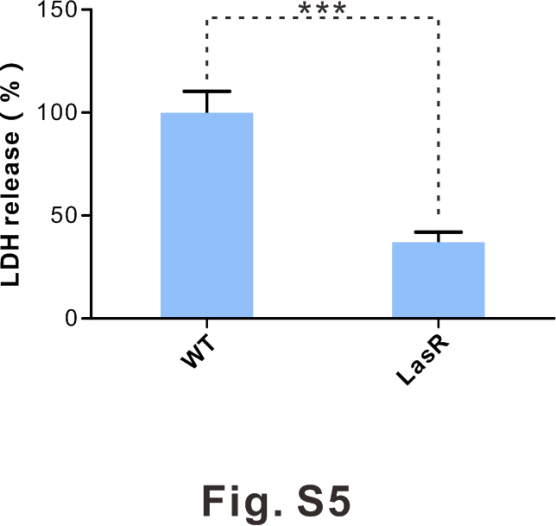


**Figure S6. Cell cytotoxicity of assayed strains.** The cytotoxicity of *P. aeruginosa* PAO1 wild-type (WT) and LasR-null mutant strains were assessed by infecting CHO cells at a multiplicity of infection (MOI) approximately 5.0. The cytotoxicity effect was estimated by the release of lactate dehydrogenase (LDH). Data are presented as means ± SD (*n* = 5). Statistical significance was determined by *t*-test analysis. *** *P* < 0.01. The experiment was repeated at least three times with similar results.
